# Clinical Trial Simulation: Planning With the OCTAVE Framework, Implementation and Validation Principles

**DOI:** 10.1002/sim.70449

**Published:** 2026-03-16

**Authors:** Kim May Lee, Babak Choodari‐Oskooei, Michael J. Grayling, Peter Jacko, Peter K. Kimani, Aritra Mukherjee, Philip Pallmann, Tom Parke, David S. Robertson, Ziyan Wang, Christina Yap, Thomas Jaki

**Affiliations:** ^1^ Department of Biostatistics and Health Informatics Institute of Psychiatry, Psychology and Neuroscience, King's College London London UK; ^2^ MRC Clinical Trials Unit at UCL University College London London UK; ^3^ Statistics and Decision Sciences Johnson & Johnson High Wycombe UK; ^4^ Lancaster University Lancaster UK; ^5^ Berry Consultants Abingdon UK; ^6^ Warwick Medical School University of Warwick Coventry UK; ^7^ Population Health Sciences Institute Newcastle University Newcastle UK; ^8^ Centre for Trials Research Cardiff University Cardiff UK; ^9^ MRC Biostatistics Unit University of Cambridge Cambridge UK; ^10^ Statistical Sciences Research Institute University of Southampton Southampton UK; ^11^ Clinical Trials and Statistics Unit The Institute of Cancer Research London UK; ^12^ University of Regensburg Regensburg Germany

**Keywords:** adaptive design, clinical trial simulation, complex innovative designs, computation, graphical tools, master protocol

## Abstract

The adoption of complex innovative clinical trial designs has steadily increased in recent years. These are trial designs that have one or more unconventional features—often resulting in multiple stages—with the goal of improving on conventional single‐stage, fixed‐setting designs in terms of efficiency, for example, by reducing the required sample size or the time to establish findings about an intervention. The motivation for these designs may not be difficult to follow, but their set‐up and implementation is usually more challenging. Statistical properties of these designs can also be difficult to compute. Clinical trial simulation (CTS), which uses software to generate artificial data for learning, can be conducted to identify the (optimal) setting of a clinical trial, evaluate the design's statistical properties under some hypothetical scenarios for sensitivity analysis, and compare different design set‐ups and data analysis strategies, all of which contribute to a better understanding of the value of unconventional features before implementing the design in an actual clinical trial. Existing literature on simulation primarily focuses on the evaluation of statistical analysis methods, with less attention on the detailed specification and planning of CTS. This tutorial presents a new framework, called OCTAVE, for outlining the details of CTS, provides practical recommendations for their implementation, and addresses key computational considerations. The target audience is trial statisticians who are involved in designing and analyzing clinical trials. This tutorial covers a range of complex innovative designs, without the expectation that readers are familiar with the mentioned examples.

## 1 Introduction

Complex innovative clinical trial designs have seen a surge in popularity in recent years, further accelerated by the research response to the COVID‐19 pandemic [1, 2, 3, 4, 5]. Similarly to the US Food and Drug Administration's (FDAs) guidance [6], we define a complex innovative design as a trial set‐up that has one or more unconventional features (such as adaptive decision making [7, 8], multiple experimental arms, multiple subpopulations, non‐standard endpoints, potential external disruptions, time effects, etc.) and for which, often, closed‐form mathematical expressions (e.g., for sample size calculation or unbiased effect size estimation) are not available. This includes study designs with master protocols (e.g., platform, basket and umbrella trials) [9, 10, 11, 12], multi‐arm multi‐stage designs [13, 14, 15, 16, 17], personalized randomized controlled trial designs [18, 19], or adaptive sequential multiple assignment randomized trial (SMART) designs [20, 21]. The definitions of these designs are available in Supplementary Document I.

The benefits of complex innovative designs in terms of efficiency in comparison to conventionally designed trials have been described at length [7, 8, 22]. The motivation for complex innovative designs may not be difficult to follow, but their set‐up and implementation is usually more challenging. The operating characteristics of the design, such as control of false positive results, the required duration to evaluate an intervention, or the optimal sample size, are often not easy to compute. Even for designs for which closed‐form mathematical expressions for some metrics are available, it may often be desirable to evaluate or characterize other metrics, such as the distribution of the required sample size, bias of estimators [23, 24], and the effect of observational delay [25, 26] or drop‐outs. In some cases, exact analytical expressions may only work well asymptotically, whereas trialists will be much more interested in finite‐sample (or even small‐sample) properties of a design. It is in these settings that simulation plays a fundamental role in allowing the estimation of metrics that cannot be calculated analytically. In the process of designing a trial while consulting with different stakeholders, it may also be valuable to undertake “what‐if” analyses, illustrating one or more examples of the trial's potential progress over time, fostering discussion about the trial's features and their implications in typical and less typical trial realizations [27, 28].

A simulation study uses a computer program to learn about different situations to understand what might happen, instead of doing a real‐world experiment [29]. The use of simulation in the context of clinical trials is not something new; several works have also discussed this topic at length. For example, a review [30] was conducted to assess the use of clinical trial simulation (CTS) in the development and clinical use of specific drugs, with respect to methodology, applications, and lessons learned from CTS. The planning of a simulation project in drug development had also been discussed from a high‐level view, from setting up the simulation team to execution of the simulation project [31]. Some insights from the industry perspective on the development of the report of a simulation study were shared [32] in view of the importance of utilizing CTS in the design and analysis of adaptive trials, as recognized by the US FDA [33].

In the literature, a tutorial paper [34] has provided a structured approach for planning and reporting simulation studies that evaluate statistical methods, with little attention to trial designs that are more complex than a two‐arm parallel group design. It was also not those authors' intention to guide readers in specifying the data generating mechanism, as mentioned in their Section 3.2. However, the specification of a data generating mechanism plays a key role in learning about the operating characteristics of a trial design, which is defined by a combination of design features and underlying assumptions. This limitation may make it difficult to set up a simulation study to investigate design options and/or data analysis strategies for clinical trials.

We therefore aim to complement existing literature about simulation studies [34, 35, 36, 37, 38, 39, 40, 41, 42, 43, 44] by providing guidance on setting up simulation studies that involve complex innovative trial designs. Our target audience is trial statisticians needing to conduct simulations to evaluate properties of their planned trial design and/or data analysis strategies, for example, when applying for funding or devising a statistical analysis plan [45, 46, 47, 48, 49, 50]. Statisticians planning to perform a simulation study would typically not have a deep understanding of the implications and interactions of the unconventional features of the trial design, so developing a simulation study in a systematic way as suggested in this paper should maximize the benefits of such an exercise. Statistical methodologists may adapt the suggestions here when evaluating novel design and analysis strategies.

The remainder of the paper is organized as follows: in Section 2 we briefly review some key statistical aspects of clinical trial designs; in Section 3 we describe the idea of CTS, some terminologies and the process of planning an actual trial with a complex design; in Section 4 we propose a framework, namely OCTAVE, for planning the details of CTS; in Section 5 we discuss issues related to the actual computation, such as the use of pseudocode, data generation, validation of code, efficiency in implementation and approximation approaches; in Section 6 we provide recommendations related to practice, such as simulation tasks management, graphical tools for presentation of simulation results, presenting the result of CTS to stakeholders, and reporting CTS in a grant application; Section 7 concludes. A thematic glossary of technical terms, organized by design names, statistical concepts, and simulation aspects, is provided in Supplementary Document I; two illustrations of OCTAVE are presented in Supplementary Document II; surrogate modeling technique with Gaussian process is described in Supplementary Document III; suggestions on the practice of making the computing code open‐access is available in Supplementary Document IV.

## 2 Clinical Trial Design: The Statistical Aspects

The PICO (Population, Intervention, Comparator, Outcomes) [51] or PICOTS (Population, Intervention, Comparison, Outcomes, Timing, and Setting) [52] framework and the ICH E9(R1) estimand framework [53, 54] assist trialists in formulating research questions that can be answered by clinical trials. Specialist clinicians, pharmacometricians, patient representatives, trial managers, database managers, and trial statisticians are typically involved in establishing the aim of a trial and the details of the study. There is no formal framework or guidance to decide what specific type of trial design is appropriate to implement for a question at hand. Nevertheless, any clinical trial design should consider the available scientific evidence (both on the disease itself and existing data) to efficiently and appropriately address the corresponding research questions. One may consider designing a trial as an iterative process that consists of two phases: conceptual planning and implementation [55]. CTS can be conducted to provide an initial understanding of the underlying trial design concepts, data collection and analysis processes, and the expected trial outcomes.

Here we briefly describe some key statistical concepts that are related to clinical trial design. This includes a review of randomization, sample size calculation, error rates, and statistical decision rules. It is not our intention to describe the process of identifying a trial design for implementation. Interested readers can find more details in existing work [56, 57, 58] and the references therein.

### 2.1 Randomization

The choice of randomization method [59, 60, 61] is a key component of any trial design. Such methods are used to minimize selection bias and ensure that the data of the randomized groups are comparable with respect to unobserved confounding factors. Randomization involves assigning patients at random to trial arms according to some defined allocation probabilities. A randomization procedure specifies how these probabilities are decided or computed to achieve certain goals (such as balancing key confounders between arms) and/or what allocation probabilities are implemented. Existing randomization methods can be categorized into the following broad classes [62], with allocation probabilities: (i) independent of patient data, such as in simple randomization or block randomization (the latter typically using random block sizes); (ii) depending on patients' baseline characteristics, such as in stratified randomization, minimization and other forms of covariate adaptive randomization; (iii) depending on patients' observed outcomes, such as in response adaptive randomization; (iv) depending on both patients' baseline characteristics and observed outcomes, such as in covariate‐adjusted response adaptive randomization.

### 2.2 Error Rates

In the context of statistical hypothesis testing, a type I error (false positive) is committed when the null hypothesis of no effect in a superiority trial is rejected for a truly ineffective intervention, whilst a type II error (false negative) is committed when the null hypothesis of no effect is not rejected for an effective intervention. When designing a study, the aim is to control the probability of making a type I error at a low level (often 5%), and the probability of not making a type II error (also known as the power of a study) at a high level (often 80% or 90%).

For complex innovative designs, generalizations to these error rates are typically considered. The family‐wise (or experiment‐wise) error rate, per‐comparison error rate, and false discovery rate [63] are some of the options to consider for controlling false positive errors in trials that evaluate multiple interventions and use statistical decision rules to allow for early stopping or other adaptations. Conjunctive power, disjunctive power, and per‐comparison power [64, 65] define probabilities of not committing false negative errors when answering multiple research questions from a single study. As in conventional fixed designs, the choice of type I and II error rates to control affects the required sample size of complex innovative designs. Definitions of these probabilities are available in the glossary in Supplementary Document I. Arguments for (or against) making multiplicity adjustment when multiple interventions are compared within a single trial have been widely discussed in the literature [66, 67, 68, 69, 70, 71, 72, 73].

The above measures correspond to probabilities that are obtained under repeated sampling using the same design. For Bayesian designs, posterior (or predictive) probability of making a claim can be used instead. For example, the counterpart of the type I error rate is the posterior probability of erroneously approving an ineffective intervention [74]; the counterpart of power can be the probability of appropriately approving a safe and effective intervention [74], or assurance (which is the prior expectation of the power, averaged over the prior distribution for the unknown true treatment effect [75]), or the expected power (which is a weighted average of the probability to reject a null hypothesis in the relevance region of treatment effect parameter). Interested readers are referred to Kunzmann et al. who clarify the Bayesian perspective of error rate measures for sample size calculation [76].

### 2.3 Sample Size Calculation

Sample size calculation [77, 78] depends on the study aim (e.g., demonstrating superiority, non‐inferiority, or equivalence), randomization method, error rates, (nuisance) parameter(s) of the outcome distribution, target effect size, decision rules for design modification and statistical inference, the number/proportion of missing values, and prior parameter(s) (when a Bayesian design is considered). For a two‐arm fixed design that uses either a simple randomization method or a covariate‐adaptive randomization method, there exist closed‐form sample size formulae for the commonly considered types of outcome, such as continuous, binary, count and survival endpoints. Examples of more complex types of endpoints include ordered categorical, composite, and hierarchical composite endpoints (e.g., for win ratio analysis), for which sample size calculation may require analytical calculation by approximation or optimization by conducting CTS. A more complex design may consider co‐primary or multiple primary endpoints to answer multiple primary research questions simultaneously. Others yet may consider the presence of surrogate or intermediate outcomes and/or repeated measurements on endpoints to reduce the required sample size [13].

### 2.4 Statistical Decision Rules

Statistical decision rules guide trial activities and decision‐making in a systematic way. At the end of a trial, they are used to answer the question at hand, based on an analysis of the outcome data. Especially in complex innovative designs with adaptive elements, statistical decision rules also play a key role during earlier stages of the trial, particularly in guiding adaptations. Such adaptation rules inform when and what elements of the trial are to change based on accrued data. They are pre‐specified at the design stage to ensure that the integrity of the study is maintained and so the impact on false positive and negative error rates can be well understood.

Interim analyses of the accrued data generate evidence to help guide decisions in line with the pre‐specified adaptation rules. The timing of interim analyses, or the “decision points,” may be specified in terms of the number of participants with observed outcome data (e.g., 50% of the total recruitment target) or the level of statistical information accumulated (e.g., 50% of the anticipated total number of events in a trial with a time‐to‐event endpoint) or according to calendar time (e.g., every 6 months). Interim analyses may trigger changes to various aspects of a trial [79, 80, 81]; examples include stopping recruitment, increasing sample size, adapting dosage (mainly for phase I and phase IIa studies), discontinuing intervention arms, modifying allocation probabilities to arms, changing endpoints, the management of existing participants (e.g., non‐responders in SMARTs are re‐randomized to other arms), and the management of trial progress (e.g., whether it is beneficial to add arms to an existing multi‐arm design [82]). Statistical rules may also limit the maximum allowed number of concurrently active arms for some complex innovative designs, and govern how delayed outcome measurements are handled at interim analyses.

## 3 Clinical Trial Simulation (CTS): Key Idea and Some Terminologies

CTS is a computational tool that uses software to generate artificial data to explore different aspects of clinical trial design, analysis methods and/or underlying factors, particularly in situation where such investigations cannot be performed analytically, as is the case with many complex innovative designs. A trial design is a structured plan that describes how a study will be conducted to address clinical research questions. Here, we refer to a trial design as the statistical and numerical aspects of the plan (specific examples are given in Section 4.3).

We define an “underlying factor” as a variable that influences trial design and conduct but has the following features:

- It is related to trial design and/or conduct.
- Its true properties/characteristics are unknown to investigators at the time of planning.
- The properties/characteristics cannot be altered by human intervention.

Although the latent properties/characteristics of these factors of the target population or disease cannot be controlled, they influence key statistical properties such as power, bias, and precision—further details and examples are given in Section 4.2. In Fisher's terminology, many of these “underlying factors” are *ancillary*—features whose distributions do not depend on the key model parameters that we aim to estimate but that govern the precision and robustness of the experiment [83]. Understanding their role is essential for robust trial planning and interpretation.

We refer to analysis methods as statistical techniques and procedures used to evaluate accumulating trial data and estimate the (average) treatment effect. These methods support inference and guide key decisions, such as whether to stop or continue randomization to a particular research intervention. All analysis methods rely on underlying assumptions regarding the data or the data generating process. Sensitivity to the assumptions can be assessed by simulation.

Broadly, CTS can be conducted to optimize the specification of a trial design, evaluate the design's statistical properties, and compare different design set‐ups and data analysis strategies, thus contributing to a better understanding of design parameter choices, assumptions about underlying variables that cannot be influenced by the trialists, and the value of unconventional features, before implementing the design in an actual clinical trial. Table 1 lists potential objectives of conducting CTS in general terms, where we consider the trial design, underlying factors, and analysis methods as the components that can be investigated using CTS. There we also provide specific examples from existing work in the literature. In the table, we do not include the assessment of the underlying assumptions of analysis methods for ease of exposition.

**TABLE 1:** Some project objectives that involve CTS. The last column presents specific examples from the existing literature.

Objective of conducting simulation	Trial design	Underlying factors	Analysis method	Illustrative example
Examine the properties of a design and analysis strategy under a given set of assumptions	Fixed	Fixed	Fixed	Examining the operating characteristics (type I error rates and power) of a group sequential design with a Pocock stopping boundary for normal outcome data with unknown variance [84].
Test multiple analysis methods under a given design and a set of assumptions	Fixed	Fixed	Vary	Assessing the performance of a proposed method of estimating the success probability through an augmented binary approach (for a composite endpoint using continuous data from tumor responses) [85], against the available analysis methods of estimating success probability from binary data only or logistic regression and Karrison's method, for a phase II two‐arm RCT with composite endpoints.
Assess the sensitivity of a design and analysis strategy with respect to the assumptions	Fixed	Vary	Fixed	Evaluating the properties of the TAILoR trial (a MAMS design with 4 arms and 2 stages) with a sequential *t*‐test assuming different delay lengths in the endpoint [86], that is, examining the expected sample size and proportion of patients who were allocated to the effective dose for scenarios with varying delay length.
Assess the sensitivity of multiple analysis methods for a given design with respect to the assumptions	Fixed	Vary	Vary	Assessing the performance measures of six different linear mixed effect models for analyzing data from a partially nested cluster randomized trial design [87]. The simulations also look at the efficiency of these different analysis strategies for different cluster sizes, number of clusters, intra‐class correlation coefficient and variance across the treatment and control arms under the null and alternative scenarios.
Test multiple designs that use the same analysis methods under a given set of assumptions	Vary	Fixed	Fixed	Investigating the performance of different adaptive randomization approaches in the RECOVERY platform trial using the beta‐binomial model [88].
Assess sensitivity of multiple designs to different assumptions with the same analysis model	Vary	Vary	Fixed	Investigating sensitivity of multiarm stepped‐wedge cluster randomized trial designs to different values of Intra‐cluster Correlation Coefficients (ICC), analyzed by a linear mixed effect model [89]. Cluster sizes and total number of clusters were held fixed to establish a baseline for efficiency. Treatment allocation sequences were varied via stochastic search to identify optimal designs. ICC was varied to determine how correlation structure impacts the relative efficiency of these identified designs compared to standard allocations.
Do all the above	Vary	Vary	Vary	Investigating the performance of different modeling approaches in platform trial with different randomization approaches under scenarios with varying time trend assumptions [90].

**FIGURE 1:**
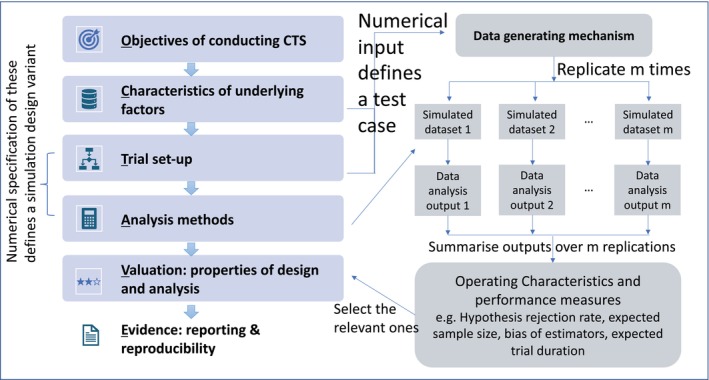
OCTAVE framework (left panel) that relates to the components in the actual simulation (right panel).

Conducting CTS for any investigation relies on the data generating process. Figure 1 shows the proposed OCTAVE framework (fully described later) for outlining the details of CTS (on the left) and the components of the actual simulation (on the right). The actual simulation involves data generation and inferential analysis. Specifically, data are generated following a trial design and a set of assumptions/conditions on the underlying factors, such that the artificial data can be analyzed by the chosen analysis methods.

We define a “data generating mechanism” as an instrument that consists of the trial design and the underlying (and, in Fisher's terms, ancillary) factors [83]. We define a *test case* as a fully specified, conditional scenario obtained by instantiating the data generating mechanism for a randomized clinical trial. It comprises (i) the trial design, (ii) the *underlying* (in Fisher's terms, ancillary) factors, and (iii) specification of analysis model, each set to concrete numerical inputs for this scenario. Synthetic datasets generated under a test case are used to evaluate *conditional* operating characteristics—performance measures given the specified ancillary configuration—without averaging across alternative configurations.

A trial design involves numerical specifications. We define a “simulation design variant” as a numerical instance of a design and analysis strategy, which can be decided by the study team. For example, a multi‐arm group sequential design with maximum likelihood estimation is the design and analysis strategy of interest. Simulation design variant one may have one interim analysis whilst variant two may have two interim analyses, to explore the role of interim analyses in this specific strategy. A comparator design and analysis strategy could be the pick‐the‐winner design [91, 92, 93] with maximum likelihood estimation, where variant one has one interim analysis and variant two has two interim analyses.

When planning a study, stakeholders can normally identify the crucial underlying factors and establish the trial design conceptually. They often may not be able to provide all the necessary numerical representations for computing the required design details, such as the sample size. Nevertheless, the conceptual information on the design and underlying factors may be sufficient for the construction of a general data generating mechanism. It is for this purpose that CTS is conducted to understand the properties of a specific simulation design variant numerically, prior to implementation.

### 3.1 Single Run/Trial Replication

Once the data generating mechanism is constructed and provided with the numerical inputs, we can simulate data on a computer. The process of simulating and analyzing data to give inferential results once is called a “single run” or “trial replication”. One trial replication mimics what data are to be collected and how the data are to be analyzed when a trial is conducted (once) in practice, that is, it represents a potential real‐life realization of a trial. In addition to the usual inference (such as estimating parameters of distributions), we can examine other information about the trial, such as: how the allocation probabilities change with the accrued data when an adaptive method is used, what statistical decisions were made at interim analyses according to the pre‐defined rules, the study duration, and the implemented sample size. While a single simulation run may be illustrative of possible trial progress, the examination cannot capture the uncertainty in the data generating process to convey how the simulation design variant performs in expectation or how likely particular types of trial progress or trial results are.

### 3.2 Repeated Runs/Replications Give Properties of a Simulation Design Variant

With multiple runs/replications, that is, repetition of the process of generating and analyzing data to obtain multiple sets of data analysis outputs, we can study the outputs to understand the properties of a simulation design variant, for example, by examining summary statistics or by visually assessing variability. Note that the repetition here is not for reproducibility purposes but for exploring the impact *caused by the stochastic elements* in the data generating process. Examples of design properties (i.e., operating characteristics [33]) include the frequency of meeting statistical decision criteria, the mean of a continuous measure (or the proportion for a binary indicator) considered in the criteria, expected trial arm duration, and expected sample size. In contrast, control of error rates, bias, mean squared error of an estimator, and the coverage of a confidence interval represent the properties of an analysis strategy (i.e., performance measures of analysis methods [34]).

As the objectives of conducting CTS are context dependent, evaluation requirements can also differ. Ultimately, the evaluation (over many trial replications) is to ensure that the proposed simulation design variant meets the required statistical properties of a study before the actual implementation, which are normally related to some, if not all, aspects mentioned in Section 2.

### 3.3 Process of Planning an Actual Trial

We now describe the process of planning a real‐world trial with a complex design. It involves multiple stakeholders in determining all key aspects of the trial, including non‐statistical aspects, for example, the ordered sequence in which interventions will be tested in a platform trial. CTS can clarify uncertainty during the process and establish the numerical aspects of the trial design and analysis strategy for implementation. In general, the steps are as follows:

1. Establish the research question(s) that the trial is intended to answer.
2. Determine the underlying factors and constraints on the proposed trial design. A “straw man” simple design, or alternative design(s) may be considered as comparator(s).
3. Select the summary measures of interest for evaluation purpose, and if needed their priority in the intervention development and evaluation program (which in pharmaceutical drug development typically consists of a series of phase 1, phase 2 and phase 3 trials leading to registration).
4. Reach consensus on the uncertainty in the assumptions on the underlying factors among stakeholders.
5. Conduct initial CTS to understand what the initial specification can potentially lead to, for example, for a simple version of the proposed design or even just the “straw man” design.
6. Present initial simulation results to facilitate reviewing all the above inputs with stakeholders.
7. Iteratively update the simulation details and report of the simulation results, working with the stakeholders to an agreed final version of the design and analysis strategy.

This suggested process aligns with the principles of good clinical trial design [55] and the concept of a “vanilla and sprinkles” design [94]. Note that the questions in step 1 correspond to the clinical study rather than the specific ones that a CTS can help answering. For example, a phase 2 trial might aim to (a) make a go/no‐go decision for going to phase 3, (b) select the dose/treatment regimen with the best chance of success in phase 3, (c) identify the population to test in phase 3, and/or (d) help determine the size of the phase 3 study. As described in steps 5–7, this process can start with a simple design and builds the complex details in an iterative way. We propose a framework to facilitate planning of CTS in the next section.

## 4 OCTAVE: A Framework for Planning CTS

We propose a new framework called, “OCTAVE”, for planning CTS, which stands for

- Objective(s) of conducting CTS
- Characteristics of underlying factors: assumed numerical representation
- Trial design(s): one or more options for evaluation
- Analysis methods: one or more approach for evaluation
- Valuation: measures to assess the value of design and analysis strategies
- Evidence: reporting and reproducibility of the CTS

Writing up the details following this framework is similar to documenting a trial project in a protocol. Figure 1 describes the framework in relation to the components of a simulation. We provide two illustrations of OCTAVE in Supplementary Document II: (i) Bayesian adaptive designs evaluation and (ii) fixed designs comparison with pharmacodynamic models.

### 4.1 Objective(s) of Conducting CTS

Table 1 describes some general objectives of conducting CTS. It is common that CTS are conducted in an iterative way to meet a specific objective or more than one objectives. Setting clear objective(s) of conducting CTS allows one to identify the details that can be kept constant and those that can be varied across the test cases. This identification helps with setting up the code. It will also be easier to start with one objective before investigating more than one objectives simultaneously.

Examples of specific objectives are: identify the required sample size of a trial design and analysis strategy, establish if the simulation design variants of a trial design and analysis strategy meet the error rate requirement, and study the robustness/sensitivity of simulation design variants to the assumptions on underlying factors. We include in Table 1 specific examples from the existing literature where simulation was implemented.

### 4.2 Characteristics of Underlying Factors: Assumed Numerical Representation

Ultimately, the purpose of running clinical trials is to study the unknowns about interventions. In reality, many underlying variables can affect the trial set‐up, conduct, and study findings. Investigators can select the specific underlying factors to consider in their simulation study, but unlike design parameters (discussed in Section 4.3) these factors have characteristics/properties that cannot be altered by human intervention in a real‐world trial. At the trial planning stage, investigators need to make assumptions on the numerical representation of these characteristics/properties, for example, based on historical data or meta‐analysis of relevant studies. Examples of factors that need assumptions at the planning stage include:

- Endpoints that reflect the clinically meaningful reaction to the intervention and the actual difference between treatment and control in the population
- Natural/Biological variability in how participants respond to a treatment
- Prognostic covariates that reflect the profile or case‐mix of targeted population(s) or sub‐populations of interest, and how they are statistically related to the endpoints (if at all)
- Risk factors that increase the probability of developing a disease or outcome
- Participant accrual rate
- Disease progression or time trend over the study duration
- Availability of interventions and their efficacy/effectiveness on endpoints
- Availability of concomitant treatments
- The tendency of participants to withdraw from the study, missing data types and pattern
- Non‐adherence to treatment

The variety of endpoint types that investigators can consider includes single endpoint, co‐primary or multiple primary endpoints, intermediate endpoints, repeated measurements and censored observations.

After selecting the specific type of underlying factors, the next task is to quantify their characteristics numerically. For example, hypothesize the distribution of the selected endpoint and underlying parameters for planning the sample size. CTS can then be conducted to verify the sensitivity of a design and analysis strategy to these assumptions.

We note that some of the underlying factors are pertinent to certain types of complex innovative design, for example, the presence of a time trend might be of particular concern in platform trials [90, 95, 96] or studies that employ response adaptive randomization methods [97, 98], but it is less of a concern in studies that do not add or drop arms. In CTS, we can either make explicit assumptions on the underlying factors or construct statistical models to represent their properties in the data generating mechanism, or mathematical models based on biology, pharmacology, physiology, and disease for quantifying the interactions between drugs and subjects (e.g., pharmacokinetic‐pharmacodynamic models, exposure–response models, disease models) [99]. For instance, a CTS that disregards the missing data mechanism assumes that data are missing completely at random (if there is any missingness in practice). For trials whose population of interest is subjects with a specific profile, one may simulate from a continuous distribution for a biomarker [100], or create virtual participants from mathematical and computational models [101]. Standardized parameterization may be employed in CTS when there is insufficient background information to inform the actual distributions.

### 4.3 Trial Design(s): One or More Options for Evaluation

We present design aspects that investigators can specify and manipulate, which combined with assumptions on underlying factors (which cannot be actively manipulated by the trialists) enable a data‐generating mechanism to produce artificial data for exploration.

- Randomization method to allocate subjects to study arms
- Patient follow‐up pattern (i.e., how frequently data is collected and what data is utilized for what decision‐making)
- Sample size
- Number and timing of interim analyses
- Statistical decision rules for guiding trial activities and decision‐making (at both the interim and final analyses)

In some instances, it may make more sense to specify the sample size by arm and/or stage rather than overall, where a stage may be defined as a time stratum that includes concurrent randomization to the treatments being compared [102]. For example, the number of active arms in multi‐stage designs may vary across stages, which may be fixed in advanced or random.

For platform trials where arms can be added to the study when they become available, the number of active arms may vary across stages. One may model the availability of interventions over time [103] or assume a fixed number of arms that will be added to the platform.

The randomization ratio may vary with the number of active arms as the trial progresses, or vary across sub‐populations due to intervention specific eligibility criteria (e.g., in personalized randomized controlled trial designs). For the latter, the randomization probability to the ineligible arms can be set to zero accordingly. In some instances, it may be appropriate to disregard the precise randomization procedure to be utilized and consider deterministic arm sizes, assuming implicitly that randomization always leads to the desirable arm size. This may most often be the case in larger studies testing solely in the entire study population.

Whether the exact patient follow‐up pattern needs to be specified will depend on the specific objectives and type of trial design under evaluation. Often, the fact that patient visits typically conform to some prespecified schedule is ignored when performing CTS. For example, in simulating progression‐free survival times in an oncology setting, it is common to disregard the fact that the exact progression time cannot be observed, owing to an inherent expectation that the impact of this on power will be small as it should impact all treatment arms equally. However, sometimes it might be the case that evaluating the specific data collection plan is part of the objectives of the CTS (e.g., assessing whether additional data collection time‐points provide power gains in a repeated measures analysis).

Sometimes the number of planned interim analyses may be fixed, for example, by logistical or practical constraints. However, it is common for an assessment of the impact of the number of interim analyses on design operating characteristics to form part of the objectives of a CTS. In this case, it is typically routine to evaluate a range of numbers of interim analyses, making the selected range with reference to implications on the computational burden during the investigation stage as well as the real trial.

We do not elaborate further on specific statistical decision rules here, as they are highly context dependent. Broadly, the choices under each design specification are closely linked to the clinical research questions and the required resources. It is essential to consider the potentially higher costs and demands on funding and team expertise that these choices may entail, especially when considering statistical decision rules other than advanced randomization methods [104, 105]. Additionally, the design depends on the required inferential results of a trial. For example, when a trial aims to establish the efficacy of only one (numerically) best intervention out of multiple candidates, the pick‐the‐winner design [91, 92, 93] is a better option than group‐sequential multi‐arm multi‐stage designs, as the number of intervention arms that reach the maximum sample size is unknown at the design stage of the latter design [106].

Once the numerical details of the underlying factors and the trial design are specified, we can simulate the data. There are two ways to simulate data for investigation: simulate aggregated level or individual participant data. The former is applicable only to design frameworks that have a known sampling distribution for the aggregated‐level outcome data. We delegate the comparison between these data generation approaches to Section 5.2.

### 4.4 Analysis Methods: One or More Approaches for Evaluation

The choice of analysis methods mainly depends on the trial research questions. Hypothesis tests on treatment effects and summary statistics about treatment effects, such as odds ratios and their associated confidence intervals, are the common inference targets in trial analysis. For designs that incorporate adaptive decision rules, the summary statistics dictated in the rules are computed at interim analyses for making decisions. Some rules also involve specific analysis methods or computation of summary statistics and/or tuning parameters in a pre‐specified manner, such as group‐sequential [107, 108, 109, 110] or multi‐arm multi‐stage [111, 112] designs with covariate adjustment and response adaptive randomization with delayed or missing data [113, 114, 115, 116, 117]. We note that the interim analyses do not typically contain the same level of detail as the final analysis of a clinical trial, for example, no or fewer sensitivity analyses. Nor are they the same as those for safety monitoring purpose, for example, monitoring of serious adverse events. However, where the interim analysis suggests the trial should stop recruitment to (a) specific intervention arm(s), further analyses will almost certainly be performed based on the accrued data.

The analyst's preference and knowledge on methods and the availability of other information, for example, external data that can be utilized in Bayesian methods, can also affect the choice of the analysis methods. Nevertheless, the chosen analysis methods should be consistent with the underlying assumptions of the design, and practically implementable. For example, for multi‐arm designs with fixed equal randomization ratio and continuous outcomes, the *Z*‐test (or *t*‐test) is appropriate for controlling the familywise error rate (following multiplicity adjustment), and the sample mean difference is an unbiased estimator for treatment effects (asymptotically). These analysis methods may not be appropriate when a specific type of response adaptive randomization procedure is used for trials with small sample sizes [97], as the data from such a design are no longer independent. Alternative analysis methods such as re‐randomization tests [118] and re‐weighting of the usual *Z*‐test [119, 120] may be chosen instead, and inverse probability weighting and Rao‐Blackwellization to produce unbiased effect estimates [121], although these can be computationally intensive.

As in practice for clinical trials, it is imperative to decide the trial data analysis methods upfront, as the results can vary with the choice of the analysis methods. This principle also applies to the interim analyses. For example, it has been shown that covariate adjustment via regression models at the interim analyses of a multi‐arm multi‐stage design can lead to a different treatment selection outcome when compared to the use of a simple *t*‐test [111]. In addition to outlining the required analysis output, it will be useful to describe the details and the essential assumptions of the chosen data analysis methods, especially when these require some user‐specific parameters/inputs, for example, multiple imputation approaches for missing data and Bayesian analysis methods. This information may help with understanding the simulation results, and/or establish the relevance of the analysis method(s) to the trial design.

### 4.5 Valuation: Measures to Assess the Value of Design and Analysis Strategies

Several studies have highlighted that the benefits of complex trial designs may not always outweigh the operational and analytical complexity they introduce [8, 86, 122, 123, 124]. We recognize that CTS primarily functions as a computational tool for assessing the statistical robustness of complex design and analysis strategies. The examples outlined in Section 3.2 and other statistical summary measures can inform if a simulation design variant, that is, a numerical instance of design and analysis strategy, is methodologically sound after considering the empirical values from a reasonable number of test cases. Health economics analysis may also be incorporated into the design and analysis of complex designs in CTS when appropriate [125, 126, 127].

When assessing the value of complex design and analysis strategies relative to that of conventional designs, investigators should consider aspects related to implementation in addition to the statistical properties. We recommend quantifying non‐statistical aspects for the assessment whenever possible. For example, consider the average time to execute interim analyses and staff hours required per participant to reflect the operational complexity; additional months to approval compared with conventional designs to reflect regulatory uncertainty; and the number of staff training sessions needed before trial launch to reflect lack of familiarity with novel designs.

Regarding the statistical properties, it might be informative to consider some measures as primary and others as secondary, as they may help investigators to understand what the design and analysis strategy could potentially lead to. For example, when the objective is to evaluate a Bayesian design and analysis strategy, the primary measures may include the predictive probability of trial success and the expected sample size, whilst secondary measures may include frequentist measures such as the type I error rate and properties of treatment effect estimates.

### 4.6 Evidence: Reporting and Reproducibility of CTS

We suggest to document all the above elements before implementation of simulation for transparency. Clear CTS planning documentation should explicitly state which components vary and which remain fixed in the simulation. This supports appropriate planning of the simulation tasks especially when the number of test cases is large. We describe how one may manage the simulation tasks in Section 6.1.

Moreover, clear reporting allows others to reproduce the simulation results without relying on the actual simulation code created by the original investigations. Having a second person to repeat the coding exercise independently prior to conducting the simulation at full scale is particularly useful to minimize human error. We make further suggestions on code validation in Section 5.3.

It might be useful to record the output of single and repeated runs of a few test cases for examination, prior to running the simulation at full scale. This step confirms the inclusion of the required inferential analysis results and summary measures in the code. As CTS can only provide empirical evaluation, good practice should report the summary measures along with Monte Carlo simulation error for reference. Examining these for a few test cases may reveal potential coding errors.

Specifically, for measures that are bounded between zero and one, we can use the following formula to approximate the Monte Carlo simulation error [128]:

$$\sqrt{\frac{p\left(1-p\right)}{r}}$$

where p denotes the estimated measure and r the number of simulation runs/replications for a test case.

For continuous measures, the Monte Carlo simulation error of the mean value can be approximated by the usual standard deviation formula:

$$\sqrt{\frac{1}{r-1}\sum_{i=1}^{r}{\left(x_{i}-\bar{x}\right)}^{2}}$$

where $x_{i}$ denotes the sample of the continuous measure from the $i^{\mathrm{th}}$ simulation run and $\bar{x}$ is the mean of the sample measure. One may also report summary statistics such as the interquartile range of the operating characteristics to reflect the variability in the result, in addition to constructing a 95% confidence interval using the Monte Carlo simulation error. These summary statistics are useful for measures that may not have a symmetric distribution. We refer to section 5.3 of Morris et al. [34] for more details on how one may choose n by considering the Monte Carlo simulation error.

Lastly, reporting of the inferential results of several runs under the same test case, where each run takes a different decision with respect to the statistical rule, helps start the discussion with stakeholders to clarify what can happen in the trial. We provide more concrete suggestions on presenting simulation results to stakeholders in Section 6.3 and reporting of simulation results for grant applications in Section 6.4.

## 5 Recommendations on Computation

This section describes the role of pseudocode, simulation of datasets at individual or aggregated level, validation of code, efficiency in implementation, and approximation. These considerations help in setting up the simulation syntax of a CTS.

### 5.1 Pseudocode for Outlining the Simulation Details

Before writing the simulation code we recommend writing a high‐level description of the proposed CTS in pseudocode. Using pseudocode allows a language‐agnostic description of the proposed CTS showing its overall structure without going into detailed implementation. It serves as a bridge between the simulation plan and the actual implementation. It can

- Simplify complex algorithms by omitting a level of detail and making clear the overall coding structure and flow. The high‐level description of individual steps allows the developer to identify separate tasks that can be structured as separate functions, such as data input, randomization, interim analyses, and stopping rules;
- Allow consideration of the overall approach before becoming immersed in detail;
- Provide a description of the proposed CTS that can be reviewed by others to identify logical flaws or missing decision points before coding begins;
- Act as documentation for the code, particularly for code review.

Ideally, writing the pseudocode description helps break the simulation process down into specific steps and decision‐making algorithms. Examples of helpful pseudocode for trial design simulations are available in [41, 129]. We provide an example of pseudocode for the case illustration of OCTAVE in Supplementary Document II: Section 2.1.

### 5.2 Simulating at an Aggregated or Individual Participant Level

There are two ways to simulate data for investigation: simulate aggregated level data from known distributions, or simulate individual participant data. Some examples of aggregated level data are the stagewise standardized effects considered in a group‐sequential design without covariate adjustment, or the mean response of subgroups for adaptive enrichment designs. The simulation coding details can be simplified when simulating aggregated level data from known distributions as randomization of individual participants can be avoided here. However, this is only applicable to trial design frameworks that rely on sampling assumptions. One cannot explore aspects related to individual participants, for example, the impact of covariate adjustment on interim decisions, the impact of drop‐outs, the imputation of missing data, and certain metrics such as trial duration that rely on recruitment/enrollment.

Simulating individual participant data can inform the sampling distribution of the summary statistics of interest (with a reasonable sample size) but not vice versa. This point is pertinent to complex designs whose operating characteristics depend on multivariate distributions of test or score statistics that have complex correlation structures, which may differ under the null and alternative hypotheses. When simulating aggregated level data for evaluation, it is crucial to define these correlation structures accurately—an increasingly difficult task as the number of adaptive features grows. In contrast, simulating individual participant data inherently captures the underlying correlation structures, making it a more robust and reliable approach in complex designs. The disadvantages include increased complexity in coding, increased storage space and reduced time efficiency, when compared with the aggregated approach.

One way to simulate individual participant data is to generate them one after another, in a dynamic way that emulates real practice. Specifically, the covariates and baseline characteristics of a participant are simulated before randomization to an arm. Outcomes of the allocated arm are then generated for this participant. This approach is commonly used in simulation studies that involve adaptive randomization, or complex models for the analysis of individual participant data.

Another way is to create a database for each trial arm in advance of randomization and data analysis, following the potential outcome framework in the context of causal inference [130, 131]. This database includes the covariates (e.g., baseline characteristics) of individual participants and the treatment effect of an intervention on the primary endpoint(s) and any intermediate/surrogate endpoint(s) if they are considered in the project. Compared to the former approach of simulating individual participant data, this approach may generate more data than required, and hence requires more storage space and more complex coding to utilize the corresponding participant data accordingly. Nevertheless, this approach grants better comparability, as all the simulation design variants can be evaluated on identical participants' data, thus decreasing the Monte Carlo simulation error using the same simulation runs or, equivalently, allowing for fewer simulation runs to get the same level of Monte Carlo simulation error. An example of a study that has used this approach is the evaluation of adaptive dose‐finding designs [132].

For multi‐stage designs, the generation of dataset and inferential analysis will generally be repeated sequentially within a single simulation run, according to (a) decision rule(s) until either a stopping criterion is met or the maximum sample size or number of stages is reached.

### 5.3 Validation of Code/Algorithm

Before running a CTS, it is essential to validate the simulation code and algorithm. Validation generally involves steps for testing the simulation code and algorithm, prior to conducting the simulation study of a project at full scale. These validation steps can be thought of as a layered process which starts with the smallest building blocks and work their way up to the full simulation. Each layer should be tested and any issues resolved before moving on. Without these steps, CTS is more likely to produce misleading outputs and generate incorrect findings. The following are some key validation steps and recommendations:

- **Unit testing**:
  – Test individual components (e.g., randomization, stopping rules) in isolation to ensure each part functions correctly.
  – Apply built‐in tools to conduct the necessary checks. Most statistical packages provide such facilities. For example, *assert* and *if* statements in Stata can be used to check that specific conditions are met after each simulation step, helping to catch errors early on (e.g., ensuring the entered sample size is valid: assertsample_size>0). In R, the package *testthat* provides rich functionality for unit testing [133].
- **Implementing error checking:**
  – Include checks that stop the code and provide informative error messages as to when and why it stopped. This helps to quickly identify and fix issues during development.
- **Statistical analysis validation**:
  – Existing methods: Compare the implementation to trusted software or known results, for example, for special cases for which the results are known.
  – New methods: Hand‐check results on small datasets or compare with similar approaches. Ensure the statistical model (e.g., REML vs. ML in mixed models) matches the assumptions when applying new analysis methods. More importantly, check which analysis method is the default option in the software/program that is being used.
- **Sensitivity and edge case testing**:
  – Test extreme cases to assess the robustness of the algorithm to variations in input parameters, for example, handling very small sample sizes in early interim analyses, extremely fast or slow enrollment rates, and very high drop‐out rates. This ensures the algorithm behaves reasonably under “stress”.
- **Testing against external results**:
  – Validate against theoretical expectations or empirical results in published papers for specific simulation set‐ups. The results might not exactly match. Large discrepancies (especially if the test results look “too good”) should raise red flags.
- **Inspection of simulated data**:
  – Output and inspect simulated datasets at interim and final analyses.
  – Check that:
    1. Simulated data reflect the intended treatment effects and trial parameters, for example, accrual and drop‐out rates.
    2. Interim analysis datasets are correctly extracted. For example, verify that the number of participants and stopping triggers align with expectations.
    3. Trial progression reflects interim decisions.
- **Trial progress testing**:
  – Confirm that interim analyses occur at the right times, and that the interim decisions are taken according to the pre‐specified criteria, and the subsequent trial progress correctly takes these interim decisions into account.
  – Ensure decisions (e.g., stopping, sample size re‐estimation) are implemented as planned.
- **Integration testing**:
  – Test the full pipeline to ensure that all components work together seamlessly. For example: individual patient data generation → randomization → outcome generation → analysis according to decision rules → computation of performance measures.

Having done some validation and testing on the different parts of the simulation code, we can then validate the overall simulation output accordingly. For example, consider reproducibility of simulation results, which requires that running the simulation multiple times under the same conditions produces the same outputs, ensuring that no variability is due to coding errors. This will include setting random seeds to ensure consistent results across runs. This helps detect hidden sources of variability due to coding issues.

Finally, it is important to assess Monte Carlo error and the overall statistical validity by:

- Assessing the simulation error using Monte Carlo standard error and potentially calculating confidence intervals around performance metrics.
- Running simulations under simple settings (e.g., with a fixed design) and comparing with analytical results, for example, checking that the empirical type I error rate matches the nominal level.

Further general advice on checking a simulation study is available elsewhere [43].

### 5.4 Efficiency in Implementation: Number of Test Cases, Speed and Memory Usage

Efficient trial simulation runs should strike a balance between processing speed, memory usage, and disk space to ensure the software and computing machine can handle large datasets or numerous iterations without slowing down substantially or crashing [134]. This can be achieved through optimized algorithms, streamlined data structures, and careful management of memory‐intensive operations. For instance, using vector operations instead of loops over observations or loading only necessary data into memory can significantly improve performance, as can the incorporation of “foreign‐language” code, for example, to leverage the speed of C++ for computationally intensive tasks in a simulation written in R using the *Rcpp* package [135, 136]. Parallel processing or high‐performance computing can distribute the computational load across multiple cores or machines to reduce the time required for simulation implementation.

Computing resources and time of implementing CTS are closely related to the number of simulation design variants and underlying factors that need investigation. A project may evaluate one or more simulation design variants with the associated assumptions on the underlying factors. The total number of test cases can easily grow out of proportions that would be reasonable and practical for the eventual visualization and comparison of the simulation design variants. It is therefore advisable to start small and build the cases up.

### 5.5 Approximation Approaches

For some complex innovative trial designs, researchers employ approximation methods to reduce computation burden, instead of doing an extensive simulation study evaluation. For example, the ROSA approach considers a utility‐based criterion in a Gaussian process to help choosing an optimal set of test cases (with simulated annealing approach) for sensitivity analysis with respect to the underlying assumptions [137]; the frequentist operating characteristics of Bayesian adaptive designs can be approximated by a Gaussian process over a range of treatment effect sizes [138, 139]; or the stopping thresholds of a Bayesian design can be approximated by a Gaussian process and Bayesian optimization such that the familywise error rate is controlled at the desired level [140]. The latter has been employed in the *adaptr* R package to compute Bayesian multi‐arm multi‐stage designs with early stopping [141].

A different approximation approach has been employed in the *gsbDesign* R package [142], which allows users to evaluate the operating characteristics of group‐sequential Bayesian designs over a range of true treatment effects (which are equally spaced). Linear interpolation of the nearest evaluated parameter choices is used to approximate the operating characteristics at specified values which are within the considered range but have not been evaluated by the algorithm.

We do not propose that readers employ approximation methods in their simulations to reduce the number of test cases. The decision should depend on the purpose of the project (e.g., for regulatory review or methodology investigation) and how the approximation method(s) is/are used. We include some details on approximation methods in the Supplementary Document III for interested readers.

## 6 Recommendations on Practice

We now make further recommendations related to practice.

### 6.1 Simulation Tasks Management

When the exploration of more than one simulation design variant is needed, for example, to compare between Bayesian and frequentist group‐sequential designs [143], one may consider the simulation of each design as an independent task. The consideration of the same set of assumptions on the underlying factors in each of these tasks will allow comparisons to be made between the simulation design variants.

In some situations, it might be useful to first compare the simulation findings of a small number of test cases before deciding the details of more test cases. For example, when the varying factor of the simulation design variants is the number of trial stages for making interim decisions, one can first focus on conducting the simulation with one, two and three interim analyses, before deciding on whether to continue the exploration of simulation design variants that have more than three interim analyses or when to stop the simulation exploration.

Likewise, for the exploration of a simulation design variant, one can start with the most important set(s) of test cases, for example, null treatment effects and the least favorable parameter configuration under the alternative. The details of additional test cases may be decided based on the findings of the initial ones, if all the details have not been decided upfront.

### 6.2 Graphical Presentation and Tools

Previous works on simulation studies have provided general advice on the presentation of results [31, 34]. This includes the choice of result reporting in either text, table or figure form. Further work has discussed how to use graphics to check the results of a simulation study (e.g., outlier detection and evaluation) [43] as well as novel plots for succinctly displaying large quantities of information [144]. Discussions in these papers also apply to the case of simulation studies for evaluating complex trial designs; we refer the reader to these articles for such general considerations. Here, we emphasize several recommendations for presenting the numerical values of performance measures related to complex innovative trials.

When using a 2‐D plot, the values of the varying component of CTS may be placed on the x‐axis and the performance measure on the y‐axis. Line curves connecting discrete points should be used cautiously: for categorical components, such trends are often uninformative, whereas for continuous components, a curve reflects empirical performance across the tested range. In the latter case, points showing CTS results should be clearly displayed to indicate that a segment of line without markers represents an approximation. Values outside the tested range of the varying component should be omitted unless extrapolation is specifically intended. Truncation of the y‐axis range may be employed to clarify trends, with all truncated values explicitly reported in the figure caption.

For CTS with a limited number of test cases or few replications per case, for example due to constraints on computing resources, it is important to reflect this in all results. In particular, confidence intervals computed using the Monte Carlo errors should be indicated clearly on plots wherever possible, or in the figure or table caption if this is infeasible.

When there are many test cases, rejection probabilities, such as the type I error rate and power, are often best presented through dot‐and‐whisker plots, with additional labeling of the estimated probabilities. Such plots should also make clear the level of simulation error, so that the evidence for error inflation is clear. Alternatively, when only a small number of test cases (or selected unique cases) are considered, a tabular presentation may be more appropriate.

For adaptation rules involving discrete events, displaying event probabilities alongside other summary measures of continuous metrics can be illuminating, particularly when some events have low probabilities. An example is the promising zone approach [145, 146] where joint interpretation of sample size summaries and the associated decision probabilities provides a more meaningful finding than separate consideration.

When comparing design variants with continuous performance measures, mean values may be similar, whereas the associated variability can differ substantially. It is therefore important to examine both measures jointly. In particular, graphical displays that depict variability can reveal patterns not apparent from means alone. For example, in survival trials, mean study durations are often comparable across design variants, while the standard deviation can vary considerably. Furthermore, it might be clearer to consider design variants as the inner‐most factor when constructing plots. For example, if treatment effect, sample size, and design type are varied and all other parameters held constant in CTS, one can create subplots by either the treatment effect or sample size, or both using a multi‐column multi‐row figure, allowing the design variants to vary within subplots.

When CTS involves many varying components of trial set‐up and/or assumptions of underlying factors, identifying their relationships with performance measures becomes challenging. Modern tools for automated visualizations such as AIRSHIP [147], INTEREST [148], simsum [149], and rsimsum [150] enable results to be displayed in a multi‐dimensional view for identification of unique results (if there are any). Once key findings or trends are identified, using simpler, familiar plots (e.g., histograms and scatter plots) can aid initial understanding, before employing advanced extensions of standard plots (e.g., beeswarm or violin plots, which better depict distributional asymmetry) for more complex results. It often requires several iterations to produce informative graphics for highlighting the most relevant findings or test cases. Comprehensive guides on suitable plot types for different data are available, e.g., at https://r‐graph‐gallery.com/ and https://royal‐statistical‐society.github.io/datavisguide/. A Tutorial on Visual Predictive Checks [151] (to describe model predictions and variability) is available on https://www.page‐meeting.org/?abstract=1434.

We include some plots for the illustration in Supplementary Document II: Section 2.1.2.

### 6.3 Presenting CTS Results to Stakeholders

Where simulation studies are used as a tool for exploring trial design options for implementation, we make the following recommendations for presenting results, bearing in mind that many stakeholders are typically not statisticians. Including definitions for technical terms ensures clarity and consistency among all participants, see for example [152] for some terminologies used in group‐sequential designs.

1. Summarize the project goals, what information the project aims to deliver, and the decisions that are to be taken on the basis of its results. Describe how these affect the trial design and analysis strategy. It might be helpful to include a flowchart describing the elements involved in CTS and the process, for example, see figure 1 of Robinson et al. [153].
2. For test cases consisting of a particular simulation design variant but varying numerical representations of underlying factors, illustrate the outputs from a single simulation run. For instance, report for a specific set‐up the characteristics of the simulated data, a true negative and a false positive trial realization when there is a null treatment effect, and a false negative and a true positive trial realization when there is a positive treatment effect, respectively, and the results of interim analyses and the corresponding consequences on trial design.
3. Once the details from a single simulation run are understood, present the performance metrics that are computed over the simulation replications. Highlight the critical metrics for test cases where the assumptions of the underlying factors are important and likely to happen. For large sets of results, use graphical representation instead of tables of numerical values to explain the findings thoroughly.
4. Summarize the assumptions on the underlying factors and their impact on the simulation findings. For example, describe how the design characteristics vary with the assumed accrual rates. Where a single value was considered for an underlying factor, ensure the stakeholders are comfortable with that.

It is crucial for all involved to be open‐minded during these discussions. Additional test cases may be required for further discussion. It is also possible that the stakeholders want to consider and compare different design and analysis strategies, for example, different early stopping rules that are more conservative or more aggressive. In this case, it will be helpful to check if there are any test cases whose results are uninteresting and can be dropped from future simulation runs.

### 6.4 Reporting CTS Results in Grant Applications

When reporting a simulation study for a complex innovative trial design in a grant application (for public funding), it is crucial to present information in a manner that ensures clarity, supports robust peer review, and inspires confidence in the design's methodological rigor and feasibility. Typically found within the statistical considerations section of the grant, this component should

- **Balance technical depth and accessibility:** Provide a concise, clear summary understandable to non‐statistical reviewers, while including sufficient technical detail for statistical experts.
- **Adapt to space constraints:** Prioritize essential details in the main application and use appendices or Supporting Information for more extensive explanations, schematics, or examples. Where allowed, provide links to external resources such as project websites, code repositories (e.g., GitHub), or detailed simulation plan/results to facilitate reviewer access.
- **Acknowledge the preliminary nature of simulations where applicable:** Recognize that it may not always be feasible to present a complete and final simulation study at the grant application stage especially if the simulations are complex and require significant development time as part of the trial set‐up process. Present preliminary results that sufficiently demonstrate the trial design's feasibility, expected performance, and robustness, ensuring reviewers can evaluable its viability even if the simulations are not yet finalized.

The recommendations in Table 2 highlight critical elements and good reporting practices for a complex innovative design.

**TABLE 2:** Key recommendations for reporting a simulation study of a complex innovative design in a grant application.

Element	Recommendation
Provide a clear description of the complex innovative design [7]	Provide an overview of innovative features (e.g., adaptive rules, endpoints, statistical models). Use diagrams or flowcharts to simplify complex workflows and decision‐making processes [28].
Communicate simulation purpose	Clearly explain how the simulation supports the proposed trial design, evaluating trial operating characteristics (e.g., sample size, probability of finding the right dose) and performance measures of analysis strategy (e.g., type I error rate, power) and addressing key objectives.
Detail key simulation assumptions and parameters	State assumptions about participant populations, endpoints, prior distributions (for Bayesian methods), or operational aspects (e.g., recruitment rates). Justify these assumptions with references to existing literature or preliminary data where possible.
Outline simulation methods	Describe the number of iterations, sensitivity analyses, and performance metrics (e.g., type I error rate, power, sample size). Specify software or provide access to user‐written codes, to ensure transparency and reproducibility.
Present results effectively	Use graphs (such as heatmaps) or tables to convey findings (e.g., type I and II error rates across different test cases). Where possible, compare results to conventional designs to help reviewers trust your findings without necessarily replicating the complex design. For instance, compare a Bayesian three‐stage design with weakly informative priors to an analogous frequentist design [143], using a freely accessible package, to show similar or better performance (e.g., based on type I and II error rates) with the same sample size. Such comparisons simplify the review process and reinforce the robustness of your approach.
Address limitations	Discuss any limitations (e.g., assumptions that may need to be relaxed in practice), and outline plans for addressing these in future simulation studies if a preliminary simulation study is presented. Be transparent about limitations and the potential impact on the study's findings to maintain credibility with the review panel.
Facilitate accessibility for reviewers	Reference Supporting Information (e.g., appendices, additional figures, methodology documents). Provide links to external detailed resources (where allowed), ensuring transparency and reproducibility [46, 49].

## 7 Conclusion

We have presented the planning details of clinical trial simulation and some recommendations to consider for implementation, with a focus on complex innovative trial designs. Additionally, we have provided recommendations on key elements to consider and present in grant applications, ensuring clarity, facilitating robust peer review, and improving confidence in a complex design's methodological rigor and feasibility. It is important to note that some of these suggestions represent just one possible approach, and alternative strategies may also be valid and effective. For example, experienced statisticians may employ an iterative approach to updating the project details instead of managing the simulation tasks as described in Section 6.1. They may first invest more time and effort in ensuring that the code is correct and optimized with respect to computation time, prior to refining the project details upon examining the results of a few test cases.

A cautionary note on using simulation studies to guide clinical trial designs is that, by necessity, we model only a simplified version of the experiment as it will unfold in the real world: simulations capture key mechanisms but inevitably omit many *covert or underlying factors*—unobserved or uncontrollable ancillary influences such as recruitment variability, site heterogeneity, and protocol deviations. Consequently, the outputs we typically report (power, type I error, precision, stopping probabilities) are estimates of *average/overall performance*—that is, overall operating characteristics across the test cases we chose to simulate—rather than guarantees for the trial as it will actually occur.

To grasp the impact of covert or underlying factors, we should deliberately vary them within the simulation plan and compute *conditional operating characteristics*: performance measures *given* particular realizations of these unobserved/uncontrollable influences. It is prudent to *re‐evaluate* these factors during the trial, using their observed values to update the assessment of the design's operating characteristics. Framing results in this conditional way both reveals where the design is robust and where it is fragile, and it aligns planning and reporting with the experiment we ultimately conduct—namely, the one realized under a specific set of *covert or underlying factors*, rather than an average over imagined worlds.

Finally, robust clinical‐trial simulation requires careful attention to practicalities: reaching consensus on parameter choices (for both ancillary and non‐ancillary aspects), coordinating multiple stakeholders in planning and conduct, and reporting findings transparently. For methods to elicit expert inputs—particularly in the context of Bayesian prior elicitation—see [154, 155, 156]. For formal reporting and documentation of complex trial simulations, see [157, 158, 159]. It is paramount to align with the principles of *transparency*, *clarity*, and *reproducibility* promoted by the international SPIRIT and CONSORT guideline extensions for adaptive designs across both early‐phase [49, 50] and late‐phase trials [46]. Taken together, these resources should enable readers to design, execute, and report clinical‐trial simulation studies effectively.

## Funding

This work was supported by the National Institute for Health Research (NIHR300051 and NIHR301614), UK Medical Research Council (MC_UU_00002/14, MC_UU_00040/03, and MC_UU_00004_09), and Health and Care Research Wales.

## Conflicts of Interest

Tom Parke is an employee and Peter Jacko was an employee of Berry Consultants, a consulting company that specializes in the design, conduct, oversight, and analysis of adaptive and platform clinical trials.

## Supporting information


**Data S1:** sim70449‐sup‐0001‐Supinfo.pdf.

## Data Availability

Data sharing not applicable to this article as no datasets were generated or analysed during the current study.
