# Supplementary material for: Clinical Trial Simulation: Planning With the OCTAVE Framework, Implementation and Validation Principles
Source: Stat Med. 2026 Mar 16;45(6-7):e70449. doi: 10.1002/sim.70449 (PMC12989786; doi:10.1002/sim.70449)
Supplement: Supplementary file 1 — Data S1: sim70449‐sup‐0001‐Supinfo.pdf. [file SIM-45-0-s001.pdf]

Clinical trial simulation: Planning with the OCTAVE framework, implementation and validation principles

Lee et al (2026) Statistics in Medicine

1 Supplementary Document I: glossary

Here we describe technical terms that have been mentioned in the paper. We classify these into design types, statistical aspects, and simulation aspects.

1.1 Design types

- Complex innovative design: A trial set-up that has one or more unconventional features and for which, often, closed-form mathematical expressions for sample size calculation and estimation of treatment effects are not available.

- Adaptive design: A broad class of trial designs that allow modifications to aspects of the trial after its initiation, without undermining the validity and integrity of the trial. Group-sequential, adaptive randomization, multi-arm multi-stage, and sample size re-estimation designs are some examples.

- Sample size re-estimation: This design allows adjustment of the trial sample size based on accrued participant data on nuisance parameters (e.g., sample variance, intra-cluster correlation coefficient) and/or the treatment effect in order for the trial to achieve a pre-specified power level.

- Group-sequential design: A multi-stage trial design that allows a trial to stop early for either futility or efficacy or both, based on accrued data so far.
- Multi-arm multi-stage (MAMS): A multi-stage design that has more than two study arms.
- Drop-the-loser: A variant of multi-arm multi-stage design where the study starts with multiple arms and at each interim analysis the remaining arms are ranked, with a pre-specified number proceeding to the next stage.
- Response adaptive randomization: A randomization method in which allocation probabilities vary based on accrued outcome data.
- Platform trial: A type of master protocol that allows new treatment arms to be seamlessly added to an ongoing trial without pausing trial activities.
- Umbrella trial: A type of master protocol that aims to identify biomarker-treatment pairs for participant subgroups classified by (a) biomarker(s) of a single disease.
- Basket trial: A type of master protocol that assesses whether a new therapy works in participant subgroups classified by an identifier of related diseases.
- Personalized randomized controlled trial design: A multi-arm design that does not have a common standard-of-care arm and has multiple lists of randomization arms that account for participants who are eligible for randomization to some but not all arms.
- Sequential multiple assignment randomized trial (SMART): A multi-stage trial design that aims to identify effective dynamic treatment regimens, also known as adaptive interventions or adaptive treatment strategies.

1.2 Statistical aspects

- Statistical decision rules: These are criteria that dictate the course of an ongoing trial (e.g., stop early or not) and decision-making (e.g., declare an intervention successful or not).

- Conjunctive power: The probability that all the null hypotheses that are false are rejected.

- Disjunctive power: The probability that at least one of the null hypotheses that are false is rejected.

- Per-comparison power: The probability of rejecting one particular false null hypothesis, e.g., one that compares a particular intervention with the control treatment.

- Familywise error rate: The probability of rejecting at least one true null hypothesis, when there is more than one hypothesis to be tested or when the same hypothesis is tested multiple times at different interim analyses.

- Least favorable configuration (of the alternative): A parameter configuration of multiple treatment effects such that there is only one effective intervention.

1.3 Simulation aspects

- Clinical trial simulation (CTS): A computer program is used to generate artificial data according to a trial design and assumptions on underlying factors for an inferential analysis conducted using a specific analysis strategy.

- Underlying factor: a variable that influences trial design and conduct, whose true properties/characteristics are unknown to investigators at the time of planning, and cannot be altered by human intervention.

- Simulation design variant: Trial design and data analysis strategy with specific numerical input (for both the interim and final analyses) that would be explored in CTS.
- Data generating mechanism: Defined by a trial design and underlying factors. It simulates data once numerical inputs are specified.
- Single run or trial replication: This involves the generation of an artificial dataset and its inferential output using a computer. This mimics the conduct of a trial in practice. (Repeated runs/replications can account for the variability in the data generation process.)
- Performance measure: A numerical quantity used to assess and describe the functioning of a trial design or analysis method. Examples include metrics such as the bias and variance of an estimator, expected sample size of a trial design, power and type I error rate.
- Operating characteristics: A numerical quantify that describes the properties of a design. Examples include hypothesis rejection probabilities and expected study duration.
- Test case: Defined by a set of numerical inputs of the design variant and underlying factors.
- Pseudocode: Simplified description that illustrates the essential steps of an algorithm or a procedure in a language-agnostic manner.
- Code validation: Conducting checks on the computing code/syntax to ensure it functions as expected.
- Open source: Open source codes/software are made freely available for public use. This enables others to evaluate, validate and reproduce the results and inferences drawn using the source code as well as extend and modify it further.

2 Supplementary Document II: illustrations of OCTAVE

We provide the details of two CTS projects following the OCTAVE framework. The complexity in the first case study lies in the design, whereas in the second case study it lies in the underlying factors. We also include an example of pseudocode and some plots for the first case study.

2.1 OCTAVE: two-endpoint adaptive design

We illustrate the OCTAVE framework using a paper that aimed to identify a novel Bayesian group-sequential design for a randomized phase 2b chemoprevention study of women at increased risk of breast cancer.[1]

Objectives of conducting CTS

Objectives: to investigate the statistical design features of the trial and provide a rationale for the final choice of its design.

The varying components in this CTS are the different trial designs and the assumption on the underlying factor. The same analysis method has been used for all test cases.

Characteristics of underlying factors: assumed numerical representation

- Primary endpoint: fibroglandular volume (FGV)
- Secondary endpoint: Ki-67, a marker of cell proliferation
- Relation between primary and secondary endpoint: assume they are independently normally distributed. Assume negative mean change indicates the intervention is more effective than the control treatment.
- Drug effect considered:
  - No effect: difference of 0 for both endpoints

  - Expected effect: -17.6 for primary, -0.4 for secondary
  - Large effect: -30 for primary, -1.8 for secondary
  - Worse effect: 5 for primary, 0.2 for secondary

- Accrual rate: the weekly accrual is assumed to follow a Poisson distribution. Two levels were considered: i) average of 0.76 participants/week, ii) linearly improved rate from 0 to 0.25 participants/week for the first 13 weeks, 0.25 to 0.5 participants/week for the next 13 weeks, and 0.5 to 0.76 participants/week for the last 13 weeks.
- Participant drop-out rate: assumed overall rate of 5% and 10%, respectively, which results in missing data for both endpoints.

Trial designs for evaluation

A novel two-endpoint Bayesian two-stage design that has:

- Number of comparative arms: 2
- Randomization ratio: equal (no mention of a specific randomization method in the paper)
- Minimum sample size: 60
- Maximum sample size: 120
- Number of stages: 2
- Interim analysis: after 60 participants have had the opportunity to complete their 6-month visit
- Adaptation decision rules at interim analysis:
  - Futility stopping: posterior probabilities that the treatment is superior to the control in both the primary and secondary endpoints are each less than 0.50.

  - Success stopping: posterior probabilities that the treatment is superior to the control in both the primary and secondary endpoints are each greater than 0.9847.

- Statistical decision rules for final inference (when stopping early for efficacy or continuing to full enrollment): posterior probability of the treatment being better than control in the primary endpoint is greater than 0.9517.

Alternative design one: one-endpoint fixed design that has

- Number of comparative arms: 2
- Randomization ratio: equal (no mention of a specific randomization method in the paper)
- Sample size: 120
- Number of stages: 1
- Stopping rules: none
- Statistical decision rules for final analysis: posterior probability of treatment arm is better than the control for the FGV endpoint is greater than 0.95.

Alternative design two: one-endpoint adaptive design that has

- Number of comparative arms: 2
- Randomization ratio: equal (no mention of a specific randomization method in the paper)
- Minimum sample size: 60
- Maximum sample size: 120
- Number of stages: 2

- Adaptation decision rules at interim analysis:
  - Futility stopping: posterior probability that the treatment is superior to the control for the FGV endpoint is less than 0.25.
  - Success stopping: posterior probability that the treatment is superior to the control for the FGV endpoint is greater than 0.9913.
- Statistical decision rules for final inference (when stopping early for efficacy or continuing to full enrollment): posterior probability of the treatment being better than control for the FGV endpoint is greater than 0.9533.

Analysis methods for evaluation

Each outcome is independently modeled as a Bayesian two-sample normal distribution using weakly informative priors, conjugate for the mean and the variance parameter. For each trial replication, posterior probabilities are computed using Markov chain Monte Carlo for decision-making following the rules described above.

The output of a single replication includes the test results, the realized sample size, number of participants enrolled, study duration and between-group differences.

Valuation: measures to assess the value of design and analysis strategies

Test cases are defined by the three study designs, treatment effect models as well as different accrual and drop-out rates detailed above. Several quantities are computed based on the averages across repeated simulation of 10000 replications for each scenario:

- Expected sample size
- Power/type I error rate
- Mean number of participants enrolled

- Mean duration of the study, both unconditional and conditional on stopping early for either success or futility
- Probability of stopping at the interim analysis for success or futility
- Probability of declaring success at the final analysis
- Posterior standard deviation of group differences for the secondary outcome

Justification of the number of repeated runs is based on a margin of error calculation. Specifically, the maximum 95% margin of error is $1.96\sqrt{0.5(1-0.5)/10000} < 0.01$; for nominal type I error rate of 5%, the 95% margin of error is $1.96\sqrt{0.95(1-0.95)/10000} < 0.004$.

Evidence: reporting and reproducibility of the CTS

The original investigators did not attach a simulation study planning document. They implemented the simulations in the Fixed and Adaptive Clinical Trials Simulator (FACTS^{TM}) and constructed an OpenBUGS program for model fitting. The latter is available in the Appendix of their paper.

2.1.1 Pseudocode

Algorithm 1 shows the pseudocode for the two-endpoint Bayesian two-stage design. The input of the algorithm is

- Design parameters:
  - Maximum sample size: $N_{\text{max}} = 120$
  - Sample size at interim: $N_{\text{interim}} = 60$
  - Interim efficacy threshold: $\theta_{\text{eff}} = 0.9847$
  - Interim futility threshold: $\theta_{\text{fut}} = 0.50$
  - Final success threshold: $\theta_{\text{final}} = 0.9517$

- Assumed values for the underlying factors: weekly accrual rate, overall drop-out rate, and drug effects for $\{\mu_{\text{control,A}}, \mu_{\text{control,B}}, \mu_{\text{treat,A}}, \mu_{\text{treat,B}}\}$.

2.1.2 Graphical representation

Examples of graphical presentations of results from repeated simulation runs are shown in Figure 1 and Figure 2. In Figure 1, two possible final sample sizes for the trial are shown (90 or 120 participants), under the two considered recruitment profiles, assuming a 5% drop-out rate. A common standardized effect size is assumed for endpoints 1 and 2; the plots then show several operating characteristics as a function of this effect. Points are used to indicate the actual values of the effect considered. The results are also faceted by the recruitment profile, so that within a single plot it is easier to compare performance for the two considered final sample sizes. Variability in the estimated values of the shown operating characteristics is also indicated in the plots.

In Figure 2, the distribution of simulated study durations is shown for two possible values of a common standardized effect size for the two endpoints. Violin plots are used as the often bimodal nature of the distribution makes boxplots inappropriate.

Algorithm 1 Pseudocode for the two-endpoint Bayesian two-stage design

```
for j ∈ (1 : M_sim) do
    i ← j
    Simulate accrual, outcomes and drop-out indicator for N_max subjects.
    D_interim ← observed outcomes of N_interim
# — Interim analysis stage —
    PP_A ← P(μ̂_treat,A > μ̂_control,A | D_interim)
    PP_B ← P(μ̂_treat,B > μ̂_control,B | D_interim)
    if PP_A > θ_eff and PP_B > θ_eff then
        Stop early ← Efficacy
        Trial continues ← FALSE
        PP_primary ← PP_A
        if PP_primary > θ_final then
            Trial outcome ← Success
        else
            Trial outcome ← Failure
        end if
        i ← j + 1
    else if PP_A < θ_fut or PP_B < θ_fut then
        Stop early ← Futility
        Trial continues ← FALSE
        i ← j + 1
    else
        Stop early ← No
        Trial continues ← True
    end if
# — Final analysis stage (if trial continues) —
    if Trial continues is TRUE then
        D_final ← Observed outcomes of N_max
        PP_primary ← P(μ̂_treat,A > μ̂_control,A | D_final)
        if PP_primary > θ_final then
            Trial outcome ← Success
        else
            Trial outcome ← Failure
        end if
    end if
    i ← j + 1
end for
Calculate and return probability of success using Trial Outcome
```

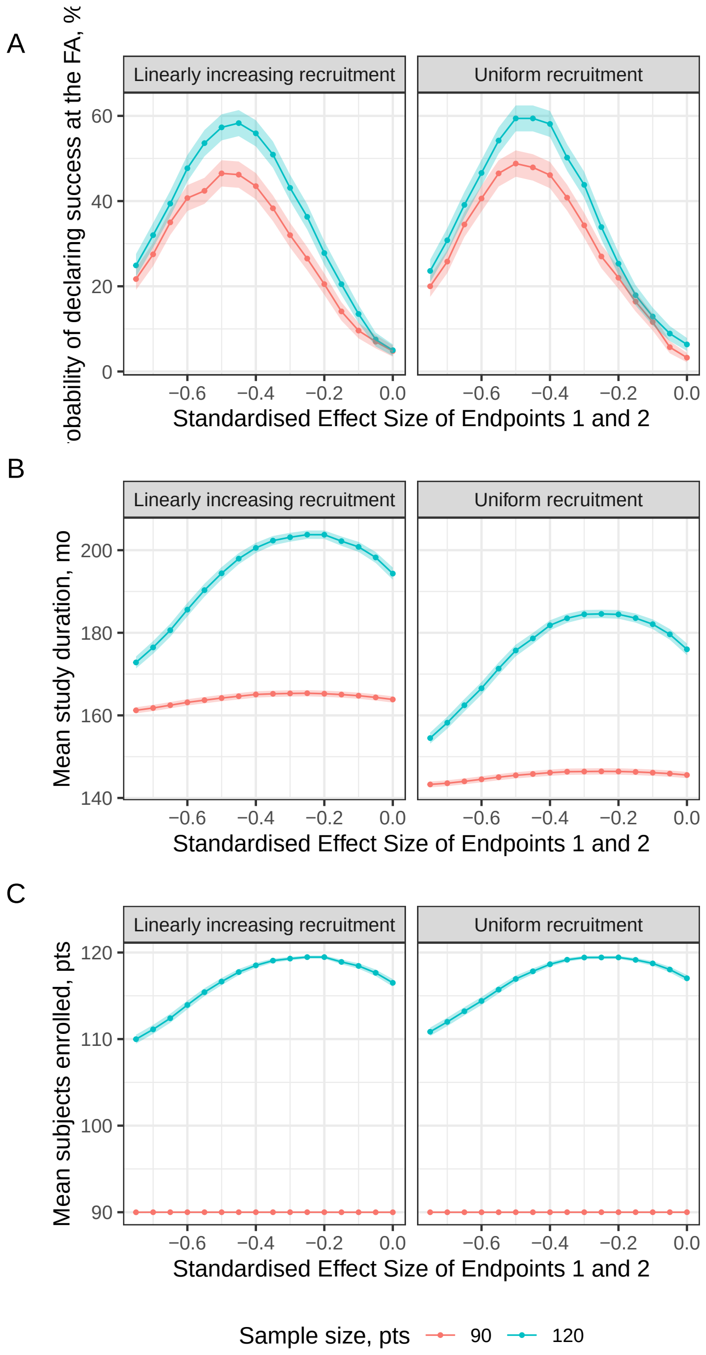

Figure 1: The estimated probability of declaring success at the final analysis (A), mean study duration (B), and mean number of participants enrolled (C) are shown for a range of test cases through dot and line curves. Specifically, the drop-out rate has been fixed at 5%, the assumed distribution of patient recruitment is then varied across the two columns of the figure, while a common standardized effect for endpoints 1 and 2 is varied across the horizontal axis, and different colors represent different maximum sample sizes. The estimated 95% confidence interval for each quantity is indicated through shading in each plot.

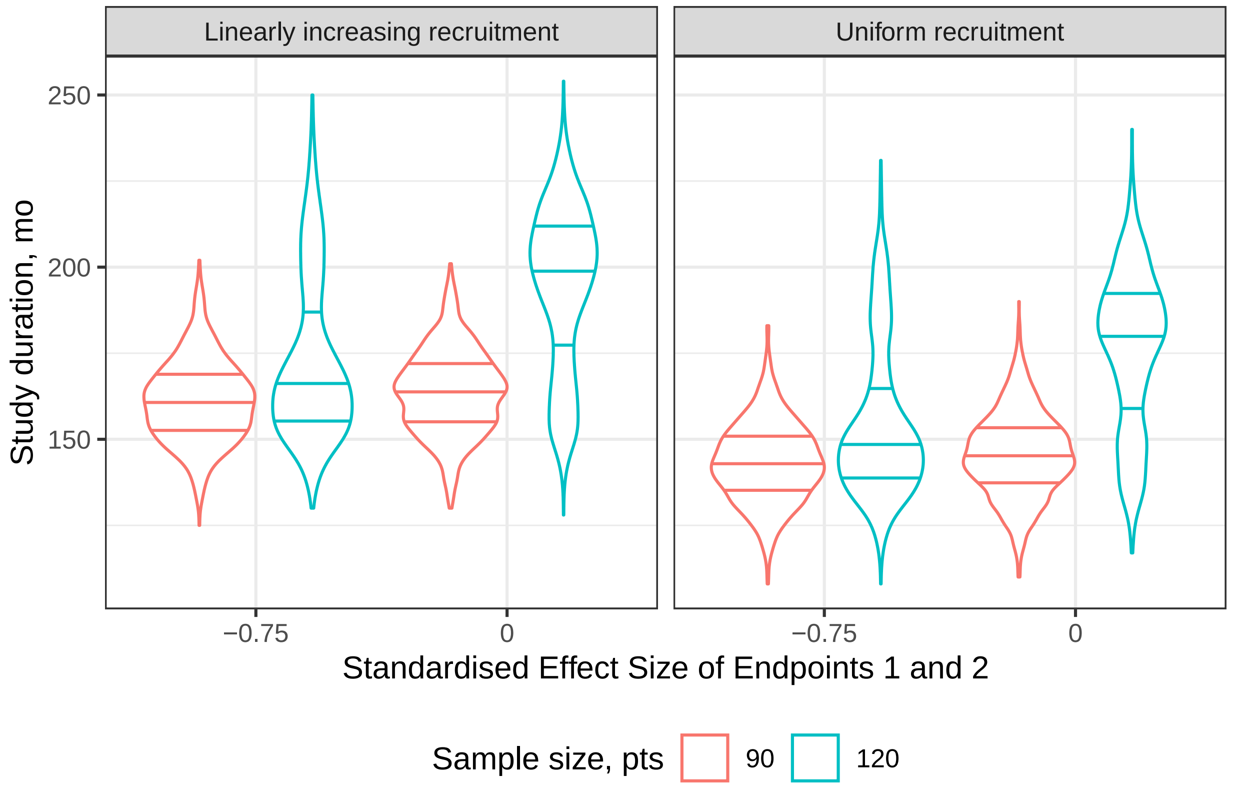

Figure 2: The distribution of the study duration is shown for several test cases through violin plots. Specifically, the drop-out rate has been fixed at 5%, the assumed distribution of patient recruitment is then varied across the two columns of the figure, while a common standardized effect for endpoints 1 and 2 is considered to be either -0.75 or 0 in each subfigure, and different colors represent different maximum sample sizes. The horizontal lines within each violin indicate the lower and upper quartile, as well as the median of the distribution.

2.2 OCTAVE: fixed design comparisons

We illustrate the OCTAVE framework using a proof-of-concept simulation study that aimed to recommend one design (out of eight) to evaluate an M1 muscarinic agonist (CI-1017), i.e., a pharmacological agent, in an Alzheimer's population[2].

Objectives of conducting CTS

- Primary objective A: to compare the power of eights designs to detect a treatment effect of a specified size
- Primary objective B: to compare design performance to differentiate a monotonic dose-response pattern from one that was U-shaped
- Secondary objective: to evaluate the bias in the drug effect size estimate relative to that assumed at pharmcodynamic equilibrium

The varying components in the CTS are the trial designs and the assumptions on the underlying factors (i.e., drug type and dose-response pattern), whilst the same analysis method was used for all test cases.

Characteristics of underlying factors: assumed numerical representation

The investigators included the following factors and assumptions in the investigation:

- Trial endpoint: Alzheimer's Disease Assessment Scale-Cognitive sub-scale (ADAS-Cog) score.
- Availability of interventions: considered safe to have multiple doses of {0, 2, 10, 25} or {0, 2, 5, 10, 15, 25} mg of CI-1017.
- Drug effects: considered five respective drug effect models/dose-response patterns, i.e., no effect, linear, hyperbolic, sigmoidal and U-shaped. Fast and slow effects were

considered for each model. These models depend on CI-1017 drug concentration at effect site. The drug concentration is assumed to follow a two-compartment model with first-order input and a lag-time, where the model parameters were obtained from fitting a model to the data of a phase 1 study.

- Disease progression: assumed a linear time course for untreated disease progression.
- Prognostic covariates: not considered in the proposed trials, but the parameters of the two-compartment model were obtained from fitting the data of a phase 1 study, which included age and smoking status in the clearance parameter.
- Missing data: assumed 1% weekly drop-out rate and a survival model for the expected percentage of patients remaining in the trial.
- Relation between interventions and trial endpoints: a population pharmacodynamic model that relates plasma drug concentrations to ADAS-Cog scores was used. It is a linear combination of a baseline ADAS-Cog value, a linear time course for untreated disease progression, a placebo effect, a drug effect and residual error that is normally distributed.

The baseline ADAS-Cog score, rate of score change, onset half-life of placebo effect, offset half-life of placebo effect, placebo effect magnitude, and active drug equilibration half-life were assumed to follow univariate independent log-normal distributions. See Table II of the investigators' paper for the numerical values of the parameters of these log-normal distributions[2].

Trial designs for evaluation

The investigators considered eight designs that vary in terms of the number of treatment sequences (consisting of four or six doses), subjects per sequence, number of treatment periods, period length (weeks) and measurements per period. These were reported in their Table IV[2].

- Randomisation method: random treatment sequences (of four or six doses).
- Patient follow-up pattern: trial length was 12 weeks for all designs except one that has 16 weeks. Please refer to Table IV of their paper for the specific number of measurements[2].
- Sample size: all designs have 60 patients except one that has 64.
- Statistical decision rules for primary objective A: declare a positive finding when at least one active dose demonstrated a trend when compared with the placebo at a two-tailed 5% significance level.
- Statistical decision rules for primary objective B: declare flat or no information regarding the shape if none of the treatment doses are significantly better than placebo at a two-tailed 20% significance level. Otherwise,
  - For the four-dose group designs, monotonicity was to be declared if the highest dose group had the best mean outcome; otherwise a U-Shaped pattern was to be declared.
  - For the six-group designs, monotonicity was to be declared if either of the two highest dose groups had the best mean.
- Statistical decision rules for secondary objective: an effect size estimate within one point (33%) of the true steady-state effect size was considered correct, independent of the outcome for objective B. The simulated estimate at the 25mg dose was used for the monotonic drug effect patterns, or the 10mg dose when the true pattern was U-shaped.
- Statistical decision rules for the trial: declare a positive finding when there is a dose-response trend (5% two-tailed significance level) and improvement of ADAS-Cog relative to placebo, at one or more active doses (2.5% one-tailed significance level).

Analysis methods for evaluation

For design selection using CTS, an analysis of variance model appropriate to each design was used to analyze the simulated data. A two degrees of freedom linear-quadratic dose trend test was implemented after fitting the model to the data. Specifically, for primary objective A, the test was conducted at a two-tailed 5% significance level. For objective B, the test was conducted at a two-tailed 20% significance level and ranking of mean outcome was used for assessing monotonicity. Effect estimates were also recorded for evaluating the secondary objective of the CTS. The output of a single trial replication includes test results and effect estimates.

For the analysis of a (hypothetical) trial that implemented the suggested design, both the 2- and 4-week measurements within each dose were included in the analysis of the ADAS-Cog subscale. A mixed-model analysis of variance was used to model the changes from baseline in total ADAS-Cog score. The model contained fixed-effect terms for treatment sequence, period, carryover effect, baseline value, and dose. Random effects were included to model the correlation between measurements within a patient, and to model the correlation between the 2- and 4-week measurements within each dose. A two degree of freedom linear-quadratic trend test was performed to test the hypotheses. The test results are the output of a single replication.

Valuation: measures to assess the value of design and analysis strategies

Test cases were defined by the study designs (eight designs) and drug effect models (two drug types with five models each). One hundred trial replications were simulated using Pharsight Trial Simulator. To identify a design for implementation, the investigators considered the following summary measures:

- For primary objective A: the percentage of trial replications that detected a drug effect, and the average of the percentages over the four active drug effect models.

- For primary objective B: the percentage of trial replications that correctly identified the dose-response shape.

- For the secondary objective: the percentage of trial replications that estimated the effect size to be within $+/-33\%$ of the true effect.

The investigators did not comment on non-statistical measures when suggesting the optimal design for implementation.

Evidence: reporting and reproducibility of the CTS

When reporting the simulation results, as the estimated power to detect an effect was always higher for the fast type drug, the investigators focused on further design performance for the slow acting drug only as it reflects the trial performance for the least optimistic scenario. Having selected the optimal design according to the summary measures, they reported the inferential results of one replication of the trial where the mixed-model analysis of variance was fitted to the data.

The investigators did not report the Monte Carlo simulation error. The code was not made available, which substantially reduces the reproducibility of the CTS.

3 Supplementary Document III: surrogate modeling technique with Gaussian process

We illustrate the idea of surrogate modeling techniques with the Gaussian process. A surrogate model acts as a stand-in for the original process/model, which often is complex. Surrogate modeling techniques can approximate computationally expensive or complex functions, enabling quicker evaluations over a specified range of parameter values.

Gaussian processes (GP) are one of the most popular surrogate modeling techniques due to their flexibility and ability to quantify uncertainty in predictions. They work by modeling

the underlying function as a distribution over possible functions, informed by some data. Formally, a GP is denoted as:

$$f(x) \sim \mathcal{GP}(m(x), k(x, x')),$$

where:

- $m(x)$ is the mean function of a multivariate Gaussian distribution, representing the expected value of $f(x)$.
- $k(x, x')$ is the covariance function (kernel), which defines the similarity between any two inputs x and x'.

The kernel plays a crucial role in defining the behavior of the GP, controlling the smoothness, periodicity, or other properties of the function. Some examples of kernel are squared exponential and Matérn kernel.

Having chosen $m(x)$ and $k(x, x')$, the process of using GP is as follows:

1. Define the range of the input variables of a simulation design variant and of the underlying factors, such as sample size and treatment effect of each arm. Each unique set of values of these variables defines a test case.

2. Run the simulation for a selected number of test cases to obtain the output of interest, such as the type I error rate or power. These cases and outputs form the dataset for fitting a GP model.

3. Fit a GP model to the data and make prediction for all the cases defined in step 1, some of which have not had output from the actual simulation. Confidence intervals can be computed for the predicted outputs. The predicted values approximate the outputs of all simulation cases.

After step 3, one can perform additional simulations to validate their accuracy by comparing the numerical outputs with the predicted values. The GP model can be re-fitted for improving the prediction accuracy, based on the initial and additional data from the actual simulations.

Alternatively, one can focus on cases with high uncertainty in the predicted outputs and use a selection strategy after the initial model fitting and prediction to pick which cases to explore next by additional simulations. This selection strategy will stop when a criterion is met. By doing so, the model fitting and prediction steps can be iteratively repeated with these additional data to improve the approximation.

Example: ROSA approach for choosing an optimal set of values of underlying factors for sensitivity analysis[3]: The aim is to select a set of numerical values of the underlying factors that best represent how some operating characteristics vary across the test cases consisting the same simulation design variant. This is achieved by optimizing a utility-based criterion as follows: a GP is first fitted to some initial data (from actual simulation runs), then used to predict the utility values for all the test cases of interest where the simulation design variant remains fixed. Algorithm 2 in the paper illustrated a case where after model fitting and prediction, additional simulations were performed to validate its accuracy by comparing the numerical outputs with the predicted values. Simulated annealing was then applied to iteratively select the set that optimizes the utility criterion, resulting in the best representative test case for the particular simulation design variant.

R code on how to use data to train a GP model and then make prediction is available in Chapter 5 of[4]. Chapter 6 presents details and coding of different criteria for the selection strategy, e.g., choose the next simulation case to maximize predictive variance, to maximize entropy and to minimize mean-squared prediction error.

4 Supplementary Document IV: additional considerations for sharing computing code

In most complex trial designs, simulations are conducted to address specific research questions. The underlying algorithms and simulation code are often reusable and can be extended for future studies or adapted by others. This section highlights key principles for improving the extensibility of simulation code and encouraging its broader use.

4.1 Open-source repositories: documentation and maintenance

Clarity and documentation: Simplicity and clarity in the code's syntax are essential for enabling users to run simulations, even if they have limited expertise in programming. Implementing consistent naming conventions for variables and functions, along with clear argument structures and standardized outputs, enhances user-friendliness and helps others follow the underlying algorithm. Additionally, minimizing unnecessary complexity in user inputs and providing clear prompts or guidelines for the required parameters can significantly reduce confusion when running simulations.

Clear and thorough documentation is vital in simulation studies to ensure the code is understandable, maintainable, and reproducible. **In-code comments** should explain the purpose of each section, function, and key decision point, allowing developers and collaborators to quickly grasp the logic and flow of the simulation. **External documentation** should provide a high-level overview of the trial's design, rationale, assumptions, and parameters, creating a clear narrative for how the simulation was developed and intended to operate.

Version control: is essential for tracking changes, managing updates, and ensuring reproducibility across different iterations of the simulation. This is particularly important when simulation code is used for methodological comparisons of different trial designs or analysis methods, or when the code may need to be revisited or rerun years after the investigation concludes – such as when results are reported to reviewers, funders, or other

stakeholders. Version control systems (e.g., Git) enable seamless tracking of code history, making it easy to reproduce exact results from previous versions and ensuring transparency in how the simulation project evolved.

Code availability and maintenance: The simulation code should be made available upon project completion and successful review. It can be published as a supplement to an existing methodology or trial protocol publication, or hosted in a separate repository. However, it is important to acknowledge the risks associated with using existing open-source simulation code. These tools often undergo limited validation due to resource constraints, particularly when applied to novel, complex trial designs. This limitation may affect the ability to compare outcomes with other trial designs or software.

4.2 Extensibility: modular design, interfaces, dialogue menu

This section is particularly relevant to software development and the creation of code at a more professional level. Extensibility enables modifications, improvements, and expansions to existing designs without requiring an overhaul of the entire codebase. A modular design separates core components—such as randomization schemes, adaptive rules, and outcome models—into distinct functions or scripts. This separation simplifies updates and additions, allowing individual modules to be modified independently. Such a structure promotes flexibility and scalability, ensuring the code remains robust as study requirements evolve.

Clear interfaces and user-friendly dialogue menus further enhance extensibility by enabling users to integrate new features without altering the core code. These interfaces act as bridges between the system and its users, streamlining the addition of advanced functionalities while preserving the integrity of the foundational framework.

An example is the *nstage* Stata command, introduced in 2009 for designing multi-arm multi-stage randomized clinical trials. Over time, this command has been updated and expanded[5]. Such extensions allow researchers to adapt the tool to evolving trial requirements, reinforcing the value of modular and flexible designs in clinical trial simulations[6]. Another

example is the R package *adoptr* that provides a customizable interface to specifying a broad class of objective functions and constraints for single- or two-arm, one- or two-stage designs with approximately normally distributed endpoints[7]. The creators also placed a great emphasis on built-in validation.

References

1. Gajewski BJ, Kimler BF, Koestler DC, Mudaranthakam DP, Young K, Fabian CJ. A novel Bayesian adaptive design incorporating both primary and secondary endpoints for randomized IIB chemoprevention study of women at increased risk for breast cancer. Trials. 2022;23(1):981.

2. Lockwood P, Ewy W, Hermann D, Holford N. Application of clinical trial simulation to compare proof-of-concept study designs for drugs with a slow onset of effect; an example in Alzheimer's disease. Pharmaceutical research. 2006;23(9):2050.

3. Han L, Arfè A, Trippa L. Sensitivity Analyses of Clinical Trial Designs: Selecting Scenarios and Summarizing Operating Characteristics. The American Statistician. 2024;78(1):76–87.

4. Gramacy RB. Surrogates: Gaussian process modeling, design, and optimization for the applied sciences. Chapman and Hall/CRC; 2020.

5. Blenkinsop A, Choodari-Oskooei B. Multiarm, multistage randomized controlled trials with stopping boundaries for efficacy and lack of benefit: An update to nstage. The Stata Journal. 2019;19(4):782–802.

6. Meyer EL, Mielke T, Parke T, Jacko P, Koenig F. SIMPLE—A modular tool for simulating complex platform trials. SoftwareX. 2023;23:101515.

7. Kunzmann K, Pilz M, Herrmann C, Rauch G, Kieser M. The adoptr package: adaptive optimal designs for clinical trials in R. Journal of Statistical Software. 2021;98:1–21.
